# miR-21-Mediated Endothelial Senescence and Dysfunction Are Involved in Cigarette Smoke-Induced Pulmonary Hypertension through Activation of PI3K/AKT/mTOR Signaling

**DOI:** 10.3390/toxics12060396

**Published:** 2024-05-29

**Authors:** Bin He, Binxia Shao, Cheng Cheng, Zitong Ye, Yi Yang, Bowen Fan, Haibo Xia, Hao Wu, Qizhan Liu, Jinsong Zhang

**Affiliations:** 1 Department of Emergency, Jiangsu Province Hospital, The First Affiliated Hospital of Nanjing Medical University, Nanjing 210029, China; hbhbwywy34@sina.com (B.H.); drwuhao@njmu.edu.cn (H.W.); 2 The Key Laboratory of Modern Toxicology, Ministry of Education, School of Public Health, Nanjing Medical University, Nanjing 211166, China; shaobinxia@126.com (B.S.); chengc1003@163.com (C.C.); 19190112@stu.njmu.edu.cn (Z.Y.); njmuyy@outlook.com (Y.Y.); fanbowen0908@163.com (B.F.); haiboxia346@163.com (H.X.); 3 Department of Emergency, Nanjing Drum Tower Hospital, The Affiliated Hospital of Medical School of Nanjing University, Nanjing 210008, China

**Keywords:** pulmonary hypertension, cigarette smoke, miR-21, senescence, endothelial dysfunction

## Abstract

Smoking is a pathogenic factor for pulmonary hypertension (PH). Our previous study showed that serum miR-21 levels are elevated in smokers. miR-21 is considered as engaged in the PH process; however, its mechanisms remain unclear. In this investigation, we found that in the lung tissue of smoking-induced PH patients, the levels of miR-21 and aging markers (p21 and p16) were upregulated, and the function of pulmonary vascular endothelial cells was also impaired. Exposure of mice to cigarette smoke (CS) for four months caused similar changes in lung tissues and increased pulmonary arterial pressure, which were attenuated by knockout of miR-21. Further, human umbilical vein endothelial cells (HUVECs) exposed to cigarette smoke extract (CSE) revealed upregulation of miR-21 levels, depression of PTEN, activation of PI3K/AKT/mTOR signaling, an increase in senescence indexes, and enhanced dysfunction. Inhibiting miR-21 overexpression reversed the PTEN-mTOR signaling pathway and prevented senescence and dysfunction of HUVECs. In sum, our data indicate that miR-21-mediated endothelial senescence and dysfunction are involved in CS-induced PH through the activation of PI3K/AKT/mTOR signaling, which suggests that selective miR-21 inhibition offers the potential to attenuate PH.

## 1. Introduction

Smoking has become a public health issue and is associated with chronic pulmonary diseases [1]. Pulmonary hypertension (PH) induced by exposure to cigarette smoke (CS) is classified as being in PH group III (resulting from lung disease and/or hypoxia) according to the World Health Organization [2]. There is controversy whether CS-induced PH is secondary to chronic obstructive pulmonary disease (COPD). The early literature suggested that emphysema destroys the pulmonary vascular bed, resulting in PH [3]. However, direct correlations between emphysema and PH have not been found in studies [4]. Exposure of guinea pig to CS causes emphysema and capillary bed destruction, but these changes are not related to increases in pulmonary artery pressure [5]. Mechanisms involved in PH include pulmonary vasoconstriction and vascular remodeling, which are present not only in COPD patients but also in smokers with normal respiratory function [6]. These results promote the hypothesis that smoking triggers the initiation of PH and is, at least in part, independent of emphysema.

Chronic exposure to CS promotes the senescence of pulmonary vascular cells, including smooth muscle cells (SMCs) and endothelial cells (ECs) [7]. Accumulated senescent ECs are present in pulmonary vessels of PH patients or animal models, and removing senescent pulmonary ECs attenuates manifestations of PH [8]. During aging, pulmonary ECs become dysfunctional and produce fewer vasodilators and more vasoconstrictors, which result in pulmonary vasoconstriction [9]. Further, the dysfunction of senescent ECs contributes to the process of PH via activating proliferation of pulmonary SMCs [10]. However, the potential impact of endothelial senescence on the development of CS-induced PH has yet to be fully explored.

miR-21, an intensively studied MicroRNAs (miRNAs), is a regulator of inflammation and contributes to senescence in ECs [11]. In addition, miR-21 is upregulated in lung tissues from PH patients and engaged in the PH process [12]. Our previous study showed that serum miR-21 levels from smokers positively correlate with smoking severity [13]. Cellular senescence is thought to be an important driving mechanism in slowly progressive lung diseases, and p21 and p16 have been implicated in senescence [14]. Senescent cells have been shown to play an important role in pulmonary vascular remodeling, accelerating the migration of PASMCs [15]. In ECs, the production of nitric oxide (NO) by the endothelial nitric oxide enzyme (eNOS) is a key factor in the correlation between blood flow and the regulation of blood pressure, and reductions in eNOS and NO increase blood pressure [16]. eNOS can be phosphorylated by kinases, including AKT, PKA, PKC, AMPK and Ca^2+^/calmodulin-dependent kinase II [17]. The involvement of miR-21-mediated endothelial senescence in the pathogenesis of CS-induced PH has not been previously reported. Thus, we speculate that upregulation of miR-21 is induced by CS and contributes to the process of PH via stimulating pulmonary endothelial senescence.

To verify the hypothesis that miR-21 is associated with CS-induced PH, we (1) evaluated the correlation between pulmonary artery pressure and miR-21 levels of human pulmonary vascular specimens, (2) investigated whether and how upregulated miR-21 promotes pulmonary endothelial senescence and dysfunction after CS exposure, thereby contributing to progression of PH, and (3) determined if knockout of miR-21 attenuated CS-induced PH.

## 2. Materials and Methods

### 2.1. Human Samples

At Jiangsu Province Hospital, we enrolled 3 groups of subjects who underwent lobectomy resection for pulmonary nodules, including 12 smokers with PH, 16 smokers without PH, and 22 non-smokers without PH as controls. This study was approved by the Institutional Review Board of Jiangsu Province Hospital. Clinical features of subjects are presented in Table 1. All subjects were males of similar ages. The lack of female smokers is consistent with previous epidemiological data revealing that the smoking rate for women is much less than that of men [1]. Further, to eliminate the possible effects of estrogen on eNOS and the cardiovascular system, the non-smoker group did not include females. The definitions of smokers and non-smokers were based on the average pack-years of cigarette usage as described in our previous studies [18]. The identification of PH by echocardiography, based on the likelihood of PH, was performed before surgery, according to the guidelines of Echocardiographic Assessment of Pulmonary Hypertension. Briefly, patients were diagnosed with PH if their echocardiograms showed: (1) a peak tricuspid regurgitation velocity (TRV) > 3.4 m/s, or (2) peak TRV > 2.8 and ≤3.4 m/s and at least two echo categories of suspected PH. Patients without PH were defined as the following echocardiographic manifestations: both peak TRV ≤ 2.8 m/s (or not measurable) and less than two echo categories of suspected PH [19]. Patients without the above echocardiographic signs were excluded, because it is difficult to discern precisely the presence or absence of PH. Other exclusion criteria were the presence of chronic cardiopulmonary disease, including PH caused by non-smoking factors. Measures of pulmonary artery systolic pressure (PASP) were performed for patients with measurable TRV, because PASP was obtained using the modified Bernoulli equation, in which TRV is necessary [20]. In patients whose lung tissue removed during surgery was surgically and rapidly pathologically proven to be a lung nodule rather than a lung cancer, all lung tissue was taken from an unaffected area, as far away from the nodule as possible. For subsequent studies, specimens of lung tissue were stored in formalin or in liquid nitrogen. From individual subjects before surgery, venous blood samples were collected into tubes containing 4% EDTA and then centrifuged at 12,000× *g* for 5 min. The plasma was stored in liquid nitrogen.

### 2.2. Cigarette Smoke (CS) Exposure

Experiments were performed with wild type (WT) and miR-21 knockout (miR-21^KO^) C57BL6 mice (male, 6–8 weeks old), obtained from Nanjing University (China). Animals were treated humanely, and study protocols were approved by the animal ethics committee of Nanjing Medical University (IACUC-2209016). The mice were divided into four groups (n = 6 each): WT mice exposed to air as control, WT mice exposed to CS, miR-21^KO^ mice exposed to CS, and miR-21^KO^ mice exposed to air. Mice in CS groups were subjected to CS exposure at a concentration of 500 mg/m^3^ of total particulate matter (TPM) as previously reported [21]. Briefly, CS were generated by burning 3R4F Cigarettes (University of Kentucky, Lexington, KY, USA). Mice were exposed to CS in an exposure device (Huironghe Technology Co., Beijing, China) for 3 h, twice a day with 4-h intervals, 5 days a week, for 4 months. Measures of right ventricular pressure was performed at the end of exposure. Then, samples of hearts, lungs, and blood were procured after being killed. Blood samples were placed in tubes containing EDTA and centrifuged at 1200× *g* for 5 min. The plasma was separated and stored in liquid nitrogen.

### 2.3. Right-Heart Function Assessment

Right ventricular systolic pressure (RVSP) was assessed for mice under anesthesia. A 23-gauge needle with a pressure transducer (AD Instruments, Dunedin, New Zealand) was threaded into the right ventricular (RV) through the diaphragm after laparotomy. RVSP was recorded using a PowerLab data acquisition system (AD Instruments, Dunedin, New Zealand). To evaluate RV hypertrophy, the RV was separated, and the ratio of RV to the sum of left ventricular (LV) and septum (LV + S) was quantified using desiccated tissue.

### 2.4. Preparation of Cigarette Smoke Extract

Soluble cigarette smoke extract (CSE) was used to imitate the stimulation of CS in vitro. Briefly, a bottle containing 10 mL of endothelial cell medium (ECM, 37 °C) was filled with smoke of standardized cigarettes (3R4F), obtained from University of Kentucky (USA). Then, the solution was modified to achieve a pH of 7.4. To establish a uniform CSE quality, absorbance values between 0.9 and 1.2 for the ΔOD (A320-A540) were considered acceptable. Finally, the solution was deemed to be 100% CSE and diluted to various concentrations for use.

### 2.5. Cell Culture, Treatment and Transfection

The human umbilical vein endothelial cells (HUVECs) utilized in this study were procured from Sciencell Research Laboratories (Carlsbad, CA, USA). These cells were cultured in endothelial cell medium (ECM) supplemented with 5% fetal bovine serum (Sciencell, San Diego, CA, USA) 100 U/mL penicillin, and 100 mg/mL streptomycin (Beyotime, Shanghai, China) at 37 °C with 5% CO_2_ and were passaged at a ratio of 1:3 every 2 days. For concentration-dependent studies, HUVECs were grown in medium and treated with 2, 4, or 8% CSE for 48 h.

HUVECs were cultured on six-well plates and transfected with the specific miR-21 inhibitor/miRNA negative control (NC) using Lipofectamine 2000 (Invitrogen, Carlsbad, CA, USA), following the manufacturer’s instructions (Genechem, Shanghai, China). miRNA NC was used as a negative control. After 24 h of transfection, these cells were treated with 8% CSE for 48 h.

### 2.6. Histology and Immunohistochemistry

In accordance with the guidelines provided by the manufacturer (Solarbio Life Science, Beijing, China), lung tissue samples from animals and humans were prepared for hematoxylin and eosin (H&E) and immunohistochemistry (IHC) staining. To conduct IHC assessment, levels of p16, p21, and eNOS (Abcam, Cambridge, UK) were assessed for 10 randomly selected arteries with a diameter of 150–350 μm (mice) or 200–400 μm (humans) per sample. Quantitative IHC staining with the immunohistochemical score (IRS) system was performed as described previously [22].

### 2.7. Senescence-Associated β-Galactosidase Staining

Assessment of cellular senescence was performed using SA-β-galactosidase (SA-β-gal) staining kits (Beyotime, Shanghai, China). Briefly, the cells were fixed with β-gal staining fixative solution for 15 min, and then incubated with SA-β-gal staining solution at 37 °C overnight. SA-β-gal-positive cells (blue staining) were considered as senescent cells.

### 2.8. Nitrite Assay

Supernatants of lung tissue homogenates and cell culture media were collected and stored at −70 °C. According to the guidelines provided by the manufacturer for the nitric oxide assay kit (Jiancheng Bio, Nanjing, China), the amounts of nitrite and nitrate, as NO metabolites, were measured using an enzymatic colorimetric assay. Nitrate was reduced to nitrite by nitrate reductase. Then nitrite was mixed with developer, and the absorbance of the dye product was measured at 550 nm by use of an Infinite M200 PRO Plate Reader (Tecan Group Ltd., Männedorf, Switzerland). The results for NO production by HUVECs were expressed as NO concentrations relative to that in control cells.

### 2.9. ELISA Assays

To determine the levels of endothelin-1 (ET-1) in culture supernatants and plasma, ELISA tests were performed according to the manufacturer’s instructions. Endothelin-1 ELISA kits were purchased from Abcam (ab13303). The minimum detection of ET-1 was 0.78 pg/mL.

### 2.10. Quantitative Real-Time PCR

The isolation of RNA from HUVECs and pulmonary samples was performed using Trizol (Invitrogen, USA). The experimental procedure involved the utilization of Power SYBR Green Master Mix (Applied Biosystems, Foster City, CA, USA) and a LightCycler 96 apparatus (Roche, Basel, Switzerland) to conduct quantitative real-time PCR (RT-PCR), following the manufacturer’s protocol. U6 snRNA was used as a control to determine relative miR-21 expressions. The primers in this study were as follows: miR-21-F, *5′-ACACTCCAGCTGGGTAGCTTATCAGACTGA-3′*; miR-21-R: *5′-TGGTGTCGTGGAGTCG-3′*; U6-F, *5′-CTCGCTTCGGCAGCACA-3′* U6-R, *5′-AACGCTTCACGAATTTGCGT-3′*. The formula 2^−(ΔΔCt)^ was used to calculate relative expressions of genes.

### 2.11. Western Blots and Quantification

The quantification of total proteins obtained from cultivated HUVECs or lung tissue samples was conducted using BCA protein detection kits (Beyotime, Shanghai, China). Western blots were performed to assess protein levels, as previous described [22]. Antibodies for PTEN, p-PI3K, PI3K, p-AKT, and AKT were obtained from Cell Signaling Technology (Boston, MA, USA). Antibodies for p21, p16, eNOS, p-mTOR, and mTOR were obtained from Abcam (Cambridge, UK). An antibody for GAPDH used as a reference gene, was obtained from Beyotime (Shanghai, China). For densitometric analyses, Image J software (version 1.53, USNIH, Bethesda, MA, USA) was used to measure relative protein bands on blots. In brief, it was changing the image to 8-bit format using Image J software and adjusting the background grey scale homogenization throughout the image to eliminate the image background effect. Control lanes were set up, and finally the grey values of each lane were obtained for subsequent quantitative analysis. Following the principle of 6-parallel in vivo experiments and 3-parallel in vitro experiments, we used ImageJ to obtain the grey values of p21, p16, eNOS, PTEN, p-PI3K, PI3K, p-AKT, AKT, p-mTOR, mTOR. Considering the high expression of GAPDH, we chose GAPDH as a reference gene GAPDH [23], compared the grey values of the target gene control lanes with the corresponding lanes of the internal reference, and obtained the average values of each target gene control group average, compared all grey values of the target proteins with the GAPDH grey values of the respective lanes, and finally, compared all the counts with the average of the target genes to obtain the target protein ploidy and input the data into prism for graphing.

### 2.12. Statistical Analysis

Data are expressed as means ± SD. The paired t-tests and RM-ANOVA tests (more than two groups) was used to compare the means of multiple groups, and Dunnett’s test was used for inter-group comparisons [24]. All data presented a normal distribution. A significance level of *p* < 0.05 was deemed to indicate statistical significance. The statistical analyses were conducted using SPSS 26.0(IBM, New York, NY, USA).

## 3. Results

### 3.1. Elevated miR-21 Levels Are Associated with Endothelial Senescence and Dysfunction in Lung Tissues from Patients with CS-Induced PH

**Figure 1:**
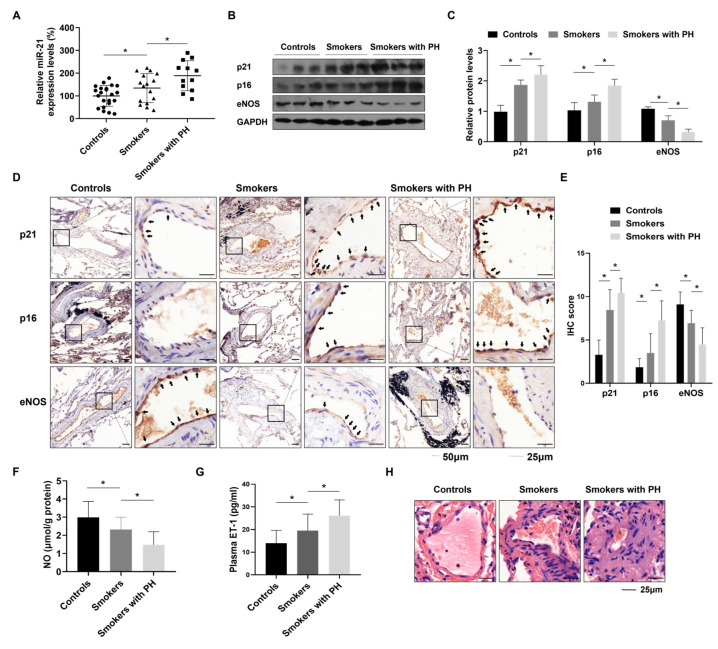
Upregulation of miR-21 is associated with endothelial senescence and dysfunction in lung tissues from smokers with PH. (**A**) miR-21 levels in lung tissues of individuals using qRT-PCR. (**B**,**C**) protein levels of p21, p16, and eNOS were measured by western blots (n = 3). (**D**) Representative immunostaining images and (**E**) the numbers of p21-, p16-, and eNOS-positive endothelial cells in human pulmonary arteries were measured using IHC (arrow: positive ECs; left bars: 50 μm, right bars 25 μm). (**F**) NO concentrations in human lung tissues were measured by an enzymatic colorimetric assay. (**G**) Levels of plasma ET-1 were determined by ELISA. (**H**) H&E staining of small pulmonary vessels (diameter: 0–150 μm, bars: 25 μm). * *p* < 0.05 compared to smokers. The arrow points to the immunohistochemistry section of the enlarged figure.

We examined the change of miR-21 in lung tissues of patients with CS-induced PH, and explored its relationship with pulmonary arterial pressure, lung aging, and pulmonary endothelial function. Pulmonary miR-21 levels were elevated in CS-induced PH patients, compared with smokers and non-smokers (Figure 1A). PASP were calculated according to the modified Bernoulli equation, in which TRV derived by echocardiography is necessary [20]. Thus, measures of PASP were performed for those patients with detectable TRV, including 12 controls, 7 smokers, and 12 smokers with PH. Among these, miR-21 levels in lung tissues significantly and positively correlated with PASP (Figure S1A). Compared with the non-smokers without PH and smokers without PH, for smokers with PH, the levels of p21 and p16, regarded as senescent markers, were high, and the levels of eNOS were low in lung tissues (Figure 1B,C). Similar results were seen with IHC analysis, in which p21- and p16-positive ECs were elevated, and eNOS-positive ECs were reduced in PH patients, compared with smokers and non-smokers (Figure 1D,E). For these patients, miR-21 levels positively correlated with IHC scores for p21- and p16-positive ECs, and inversely correlated with IHC scores of eNOS-positive ECs (*p* < 0.001) (Figure S1B–D). A hallmark of endothelial dysfunction is lack of a protective effect of NO due to decreased synthesis from eNOS [25]. In contrast, ET-1, an endothelium-derived contracting factor, is over-released in such dysfunctions [26]. In the present study, for smokers with PH, signs of endothelial dysfunction, including low NO levels of lung tissues and elevated plasma ET-1 levels, were evident (Figure 1F,G). Contrary to eNOS, ET-1 levels in patients positively correlated with miR-21 levels (*p* < 0.001) (Figure S1E). In addition, the lungs of smokers with PH showed thickened small pulmonary vessels with muscularization (Figure 1H), which is regarded as a pathological vascular change of PH. In brief, these results indicated that overexpression of miR-21, induced by CS exposure, is associated with pulmonary endothelial senescence and dysfunction contributing to PH in patients.

### 3.2. CS Induces RVSP Elevation, Right Ventricular Hypertrophy, miR-21 Upregulation, and Endothelial Senescence and Dysfunction of Lungs in Mice

**Figure 2:**
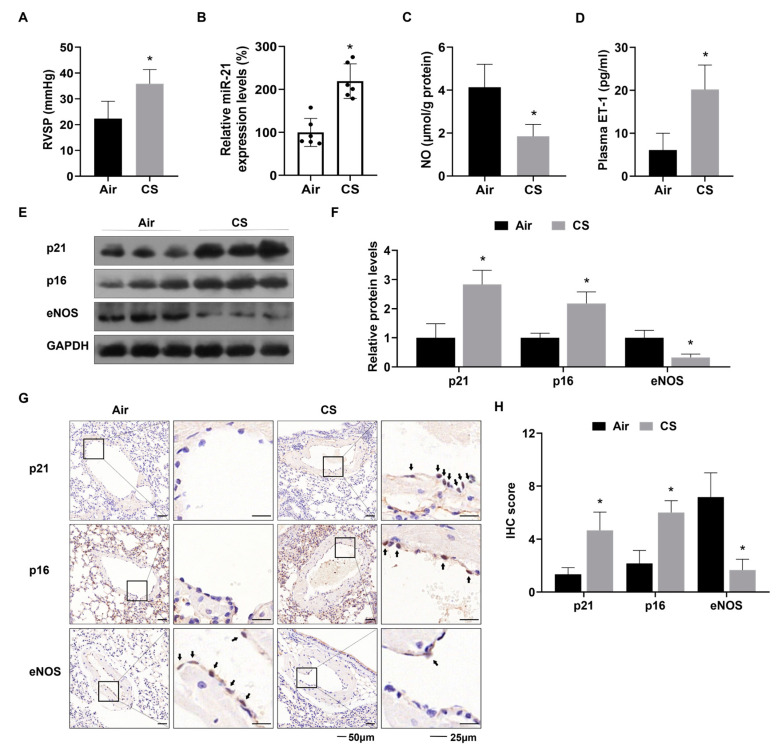
CS exposure induces increases of pulmonary artery pressure, upregulation of miR-21 levels, and senescence and dysfunction of the pulmonary endothelium in mice. (**A**) Levels of RVSP were measured in mice. (**B**) miR-21 levels in lung tissues of mice by qRT-PCR. (**C**) NO concentrations in lung tissues of mice were measured by an enzymatic colorimetric assay. (**D**) Levels of plasma ET-1 in mice were assessed by ELISA. (**E**,**F**) levels of p21, p16, and eNOS were measured by western blots (n = 3). (**G**) Representative IHC staining images and (**H**) the numbers of p21-, p16-, and eNOS-positive ECs in mouse pulmonary arteries were measured (arrow: positive ECs; left bars: 50 μm, right bars 25 μm). * *p* < 0.05 compared with mice exposed to air.

The RVSP was higher in the CS group than in the control group (Figure 2A). An increase in RV pressure tends to stimulate hypertrophy of the right ventricle wall [27]. The CS group showed an elevation in the mass ratio of RV to (LV + S) (*p* < 0.05) (Figure S2A). In addition, H&E staining of lung sections from CS-exposed mice revealed muscularization and thickening of small pulmonary vessels (Figure S2B). These findings confirmed that a mouse model of PH had been established after four months of CS exposure. Further investigation showed that expression of miR-21 was upregulated in lung tissues of mice exposed to CS versus control mice (*p* < 0.05) (Figure 2B). Reduced NO levels in lung tissues and increased plasma ET-1 levels, regarded as manifestations of endothelial dysfunction, were evident in the CS group (*p* < 0.05) (Figure 2C,D). Compared to the clean air exposure group, analysis of protein expression via western blots confirmed that levels of p21 and p16 were elevated, and levels of eNOS were lower in lung tissues of CS-exposed mice (*p* < 0.05) (Figure 2E,F). IHC staining of the pulmonary arteries showed similar results, with higher p21- and p16-positive ECs in the CS group compared with the Air group and fewer eNOS-positive ECs (*p* < 0.05) (Figure 2G,H). Our data indicate that elevated miR-21 expression and endothelial senescence and dysfunction contribute to PH in mice after CS exposure.

### 3.3. Exposure of HUVECs to CSE Upregulates miR-21 Levels, Activates PTEN-PI3K/AKT/mTOR Signaling, and Leads to Endothelial Senescence and Dysfunction

**Figure 3:**
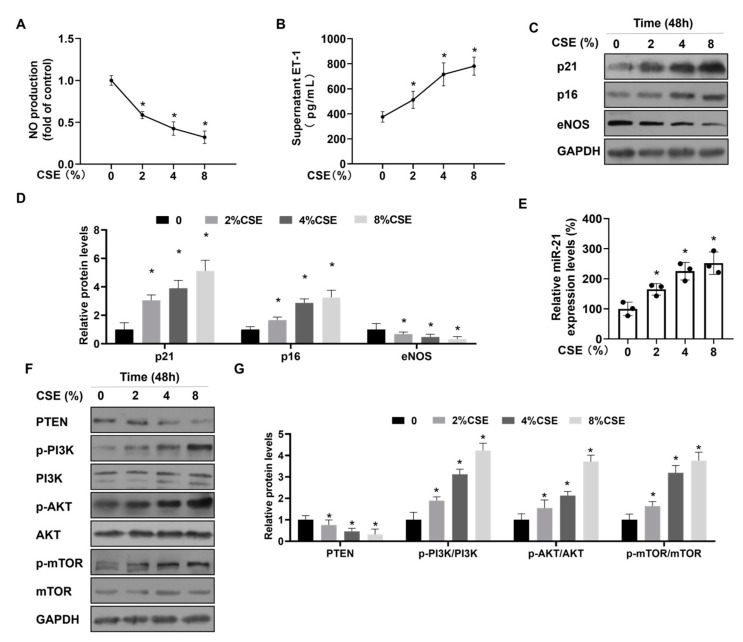
CSE causes upregulation of miR-21 levels, activation of PTEN-PI3K/AKT/mTOR signaling, and senescence and dysfunction in HUVECs. (**A**) The NO production in HUVECs was assessed by an enzymatic colorimetric assay (n = 3). (**B**) ET-1 levels in the supernatants of HUVECs were measured with ELISA (n = 3). (**C**) The protein content of p21, p16, and eNOS in HUVECs was analyzed by western blots (**D**), and levels of relative protein were measured (n = 3). (**E**) Quantitative RT-PCR was performed to determine the levels of miR-21 (n = 3). (**F**) The protein contents of PTEN, p-PI3K, p-AKT, and p-mTOR in HUVECs was analyzed by western blots, and (**G**) levels of relative protein were determined (n = 3). * *p* < 0.05 different from HUVECs not treated with CSE.

To investigate the effect of CS on senescence and dysfunction of ECs, we treated cultured HUVECs with CSE (0, 2, 4, or 8%) for 48 h. With increasing CSE exposure, the levels of NO were reduced; the levels of ET-1 increased (Figure 3A,B); and the numbers of senescent cells increased in a concentration-dependent manner (Figure S3A,B). These data indicated that CSE exposure induced senescence and dysfunction of HUVECs. Similar results were seen with western blots, in which levels of eNOS were low, and levels of the senescent markers p21 and p16 were high in HUVECs treated with CSE (Figure 3C,D). Expression of miR-21 was elevated in HUVECs treated with CSE (Figure 3E), which was similar to data for smokers with PH and mice exposed to CS. PTEN, an inhibitory target of miR-21, suppresses activation of PI3K/AKT/mTOR signaling [28,29]. After treatment of HUVECs with CSE, levels of PTEN were reduced, and levels of p-PI3K, p-AKT, and p-mTOR were increased (Figure 3F,G). PI3K/AKT/mTOR signaling is involved in the induction of cellular senescence [30]. Hence, these results indicate that miR-21 and PTEN-PI3K/AKT/mTOR signaling are involved in the senescence and dysfunction of HUVECs after CSE treatment.

### 3.4. In HUVECs, miR-21 Is Involved in the CSE-Induced Endothelial Senescence and Dysfunction via Activation of PTEN-PI3K/AKT/mTOR Signaling

**Figure 4:**
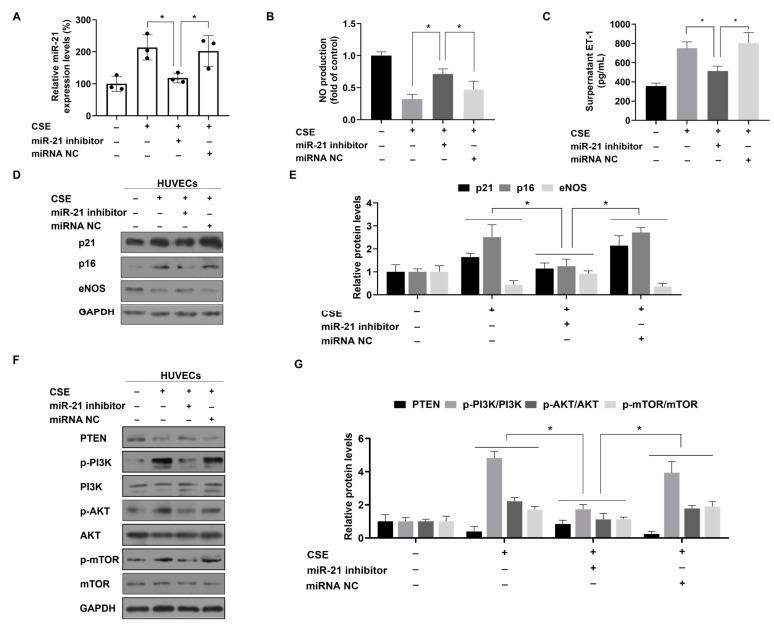
miR-21 is involved in reducing PTEN expression and activating PI3K/AKT/mTOR signaling during CSE-induced senescence and dysfunction of HUVECs. (**A**) The levels of miR-21 in HUVECs were measured with quantitative RT-PCR (n = 3). (**B**) The NO production in HUVECs were determined by an enzymatic colorimetric assay (n = 3). (**C**) ET-1 levels in the supernatant of HUVECs were determined with ELISA (n = 3). (**D**) Western blots were performed, and (**E**) relative protein levels of p21, p16, and eNOS in HUVECs were determined (n = 3). (**F**) The protein contents of PTEN, p-PI3K, p-AKT, and p-mTOR in HUVECs were analyzed by western blots, and (**G**) levels of relative protein were measured (n = 3). * *p* < 0.05 different from HUVECs treated with 8% CSE and miR-21 inhibitor.

To determine whether and how miR-21, induced by CSE, is involved in the senescence and dysfunction of HUVECs, we blocked the effects of miR-21 through transfecting an miR-21 inhibitor into CSE-treated HVUECs (Figure 4A). ELISA showed that the abnormal secretion of NO and ET-1 associated with endothelial dysfunction was reversed by inhibiting miR-21 levels (Figure 4B,C). SA-β-gal staining showed that downregulation of miR-21 suppressed the increase in senescent HUVECs induced by CSE (Figure S4A,B). Western blots showed that, for HUVECs treated with CSE, low miR-21 expression reversed the change of proteins related to endothelial dysfunction and senescence (Figure 4D,E). After downregulating miR-21 expression, we determined the levels of PTEN and components of its downstream pathways, including p-PI3K, p-AKT, and p-mTOR, in the cells. Compared with HUVECs treated with CSE, transfection of these cells with an miR-21 inhibitor showed high PTEN levels and low p-PI3K, p-AKT, and p-mTOR levels (Figure 4F,G). These results confirm that overexpression of miR-21 induced by CSE prompts senescence and dysfunction of HUVECs through modulation of the PTEN-PI3K/AKT/mTOR signaling pathway.

### 3.5. Downregulation of miR-21 Alleviates Endothelial Aging and Dysfunction and Prevents CS-Induced PH

**Figure 5:**
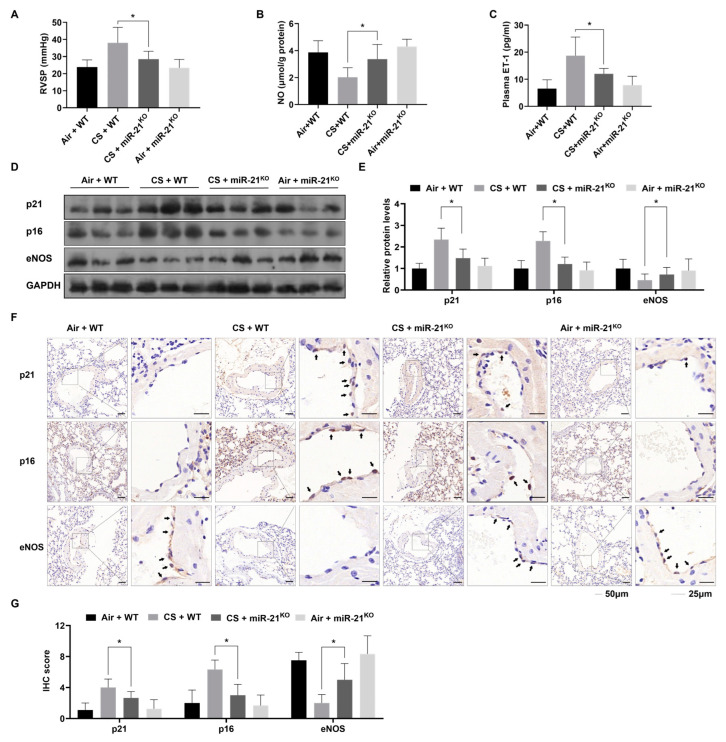
Knockout of miR-21 reduces pulmonary artery pressure and alleviates endothelial senescence and dysfunction in lung tissues from mice exposed to CS. (**A**) Levels of RVSP were measured for mice. (**B**) NO concentrations in lung tissues of mice were measured by enzymatic colorimetric assay. (**C**) Levels of plasma ET-1 in mice were measured by ELISA. (**D**) Western blots were used to analyze protein concentrations in lung tissues, and (**E**) levels of p21, p16, and eNOS were measured (n = 3). (**F**) Representative IHC staining images and (**G**) the numbers of p21-, p16- and eNOS-positive endothelial cells in mouse pulmonary arteries were measured (n = 6, arrow: positive ECs, left bars: 50 μm, right bars 25 μm). * *p* < 0.05 compared with WT mice exposed to CS.

To determine if miR-21 is involved in CS-induced PH, we used miR-21^KO^ mice. After four months of exposure to CS (500 mg/m^3^ TPM), WT mice showed an increase in RVSP and mass ratio of RV to (LV + S) which were not evident in miR-21^KO^ mice (Figure 5A and Figure S5A). Histological analysis showed that the muscularization and thickening in small pulmonary vessels was alleviated in miR-21^KO^ mice exposed to CS, compared with WT mice exposed to CS (Figure S5B). These findings demonstrated that inhibition of miR-21 expression prevented CS-induced PH and right-heart dysfunction. Further investigation indicated that CS induced endothelial dysfunction, as shown in WT mice by reduced NO levels in lung tissues and increased plasma ET-1 levels, effects that were not evident in miR-21^KO^ mice (Figure 5B,C). To confirm whether CS-induced cellular senescence was related to this process, eNOS, p21, and p16 were analyzed by Western blots and IHC. After exposure to CS, the lungs of miR-21^KO^ mice showed higher levels of eNOS and lower levels of p21 and p16, compared with WT mice (Figure 5D,E). IHC staining revealed the same results (Figure 5F,G). These data show that, for PH mice, miR-21 is involved in the CS-induced, age-related dysfunction of the endothelium.

## 4. Discussion

PH is a severe and progressive disease with multiple causes [2]. In the context of CS-induced PH, the endothelial dysfunction caused by CS is involved in the PH process [31]. A study exposing guinea pigs to CS found that chronic CS exposure induced pulmonary histopathological changes associated with COPD and PH, such as reduced endothelium-dependent vasodilation of the pulmonary arteries, complete muscularization of the small pulmonary vasculature, thickening of the lung wall, increased contractility of the main pulmonary arteries and enlargement of the alveolar spaces. Among these changes, PH and its related vascular alterations, like pulmonary vascular dysfunction and remodeling, appeared before the development of emphysema [32]. This finding coincides with our experimental results. Endothelial dysfunction occurs in the early stages of PH and is reflected by reduction in the secretion of vasodilators, such as NO and prostacyclin, and overproduction of vasoconstrictive factors, such as ET-1 and thromboxane [33]. Reduced generation of NO, due to reduced synthesis of eNOS in ECs, attenuates its protective effect against PH, including vasodilation and inhibition of proliferation of pulmonary SMCs [9,10]. ET-1, an endothelium-derived contracting factor, interacts with NO to maintain vascular tone. It is excessively produced by dysfunctional ECs and activates signaling pathways in pulmonary SMCs, promoting proliferation and vasoconstriction [26]. Plasma ET-1 levels are elevated in both PH patients and smokers [26,34]. Treatments targeting the NO and ET-1 pathways attenuate PH, and their related drugs (such as riociguat and bosentan) are clinically approved for reducing pulmonary artery pressure [35]. In the present study, downregulation of eNOS expression, reduction in NO, and increase in ET-1 were evident for smokers with PH, for mice exposed to CS, and for HUVECs treated with CSE. These results support the hypothesis that endothelial dysfunction is caused by CS exposure and is involved in PH.

Cell senescence is regarded as an irreversible growth arrest in response to various stimuli [36]. For lung cells, exposure to CS induces expression of senescence markers [37], and increased numbers of lung senescent cells are present in patients with PH [15]. Removing senescent pulmonary ECs leads to hemodynamic and histological reversal of PH [8]. These conclusions are consistent with our current data, which revealed that there were high numbers of p16- and p21-expressing ECs in pulmonary arteries from mice exposed to CS and from smokers with PH. These data demonstrated that endothelial senescence was involved in the PH process; however, its mechanism was unclear. The senescence of ECs is a cause for endothelial dysfunction [38]. The administration of anti-aging treatment to PH model mice, induced by monocrotaline, attenuates pulmonary endothelial dysfunction and manifestations of PH [39]. These data show that a dysfunctional endothelium associated with aging has a role in PH, a point supported by our results. In the present study, for ECs in vivo and in vitro, upregulation of senescent markers was accompanied by endothelial dysfunction after exposure to CS components. In conclusion, our findings suggest that senescence of pulmonary ECs contributes to the endothelial dysfunction in PH.

Overexpression of miR-21 was present in PH patients [40]; however, there is controversy about how it functions in PH. Possible reasons are that PH could be induced by various risk factors and that, for patients as well as animal models, it is related to complex mechanisms. For example, in addition to exposure to CS, PH in animal models is induced by hypoxia, SU5416, monocrotaline, bleomycin, or by surgical invasive methods [41]. It was found that overexpression of miR-21 enhanced the value-added of pulmonary artery smooth muscle (PASMC) in vitro, and knockdown of miR-21 reversed this result, which represents that miR-21 can serve as a biomarker in the development of PH [42]. Regarding smoking as a triggering factor, our results showed that miR-21 knockout attenuated the manifestations of PH in mice exposed to CS, supporting PH promotion by miR-21. We previously reported that the increase in serum miR-21 levels positively correlates with the severity of smoking [13]. Using a congenital heart disease (CHD)-induced PH model, a unique senescence-centered phenotypic shift was found in endothelial cells. In pulmonary microvascular endothelial cells (MVEC) cultured from idiopathic PH patients, shear-stress-induced DNA damage and senescence were observed. Overall, these data link senescence with the development of PH, which is consistent with our findings [43]. In the present study, we also found that miR-21 levels were elevated in lung tissues of PH, and, for cultured HUVECs, CSE exposure upregulated the expression of miR-21. miR-21 is considered to be a pro-senescence effector to ECs and is involved in age-related diseases [11,44]. These conclusions are consistent with our data, which showed that inhibition of miR-21 suppressed the endothelial senescence caused by CS. CS-induced endothelial dysfunction was reversed by miR-21 depletion, along with downregulation of senescent markers. These results demonstrate that upregulation of miR-21 contributes to CS-induced PH via promoting aging-related endothelial dysfunction.

To explore the mechanism of endothelial senescence mediated by miR-21 in CS-induced PH, we conducted experiments with HUVECs treated with CSE. Upregulation of miR-21 in HUVECs activates PI3K/AKT/mTOR signaling through depressing PTEN expression, and subsequently promotes cellular senescence and dysfunction. PTEN, a lipid phosphatase, is inhibited by miR-21 at a post-transcriptional level [28]. This is consistent with the results of our previously reported article, illustrating the regulatory relationship between miR-21 and PTEN [45]. Downregulated PTEN contributes to activation of PI3K/AKT/mTOR signaling and is engaged in multiple cellular functions [29]. PI3K/AKT/mTOR signaling is also involved in mediating cellular senescence, and blocking of this pathway, at various points, extends lifespans from yeast to mammals [30]. mTOR overactivity for mice lead to elevated lung-cell senescence, remodeling of small pulmonary arterioles, and an increase in pulmonary arterial pressure [7]. mTOR inhibitors attenuate pulmonary arterial pressures and manifestations of PH in PH patients and animal models [46,47]. These show that the mTOR pathway can be regarded as a potentially therapeutic target in PH. In the present study, our findings support the causal role of mTOR in PH, and explore the link between miR-21 and the mTOR pathway in the mechanism of CS-induced PH development.

Despite the previously unexamined mechanistic pathways explored here, we are aware of study limitations. First, human pulmonary artery endothelial cells (HPAECs) may be more optimal for implementation in this experiment than HUVECs. However, the difficulty of collection and cultivation of HPAECs hinders their adoption. The main advantage of HUVECs is that they are widely used to research endothelial mechanisms related to various diseases in diverse organs. In addition, HUVECs originating from humans have more clinical significance than those from other species. Second, right heart catheterization (RHC) is the gold standard for diagnosis of PH [2]. However, RHC is an invasive procedure and is subject to ethical limitations in this study. Therefore, we used noninvasive Doppler echocardiography, which is applied to assessing the likelihood of PH rather than directly measuring the pulmonary artery pressure [48]. According to the guidelines of the Echocardiographic Assessment of Pulmonary Hypertension, we determined PH or not for those patients with high or low probability [19], and thereby some patients with intermediate probability were excluded. Future research requires larger sample sizes and more exact diagnostic criteria, such as RHC. In addition, the number of population samples we collected for immunohistochemistry, pathology sections and western blots were insufficient to detect all indicators. In a follow-up study, we will expand the population sample size to detect all relevant indicators. Another limitation was that at the time we included the population sample, the number of people with PH was really limited in order to try to ensure some efficacy in terms of comparability between groups. In the end, there were 22 people in the control group, 16 people in the smoking group without PH, and 12 people in the smoking group with PH. In future studies, there is a need to continue to expand the sample size as much as possible to control for power of test.

## 5. Conclusions

Our results highlight the role of age-associated endothelial dysfunction in CS-induced PH. Furthermore, upregulation of miR-21 levels, induced by CS exposure, promotes senescence of pulmonary artery endothelial cells through activating PTEN-PI3K/AKT/mTOR signaling. Age-related endothelial dysfunction leads to lower NO and elevated ET-1 release, which causes contraction and proliferation of pulmonary vessels. Finally, CS triggers the development of PH through causing vasoconstriction and vascular remodeling. Our conclusions support the concept that selective miR-21 inhibition offers the potential to attenuate PH.

## Figures and Tables

**Table 1:** Clinical characteristics of subjects.

	Controls (n = 22)	Smokers (n = 16)	Smokers with PH (n = 12)
Age, years	53.8 ± 2.9	54.5 ± 2.9	54.2 ± 2.0
BMI, kg/m^2^	19.4 ± 3.9	18.5 ± 2.2	21.1 ± 4.1
Smoking history, Pack-years	0	26.0 ± 9.3 **	31.7 ± 5.8 **
LVEF, %	62.5 ± 3.0	63.6 ± 3.4	61.2 ± 3.3
Peak TRV ^#^, m/s	2.46 ± 0.34	2.40 ± 0.32	3.08 ± 0.68 **
RAD, mm	34.5 ± 3.1	35.9 ± 3.4	35.1 ± 3.2
PASP, ^#^ mmHg	29.7 ± 6.4	28.1 ± 5.8	44.6 ± 3.1 **
RVIDd, mm	30.3 ± 4.5	28.9 ± 3.2	34.7 ± 14.5 **
LVIDd, mm	48.4 ± 4.7	47.5 ± 4.9	47.2 ± 5.1
RVIDd/LVIDd, %	54.0 ± 10.2	52.4 ± 7.9	67.9 ± 7.3 **

PH, pulmonary hypertension; BMI, body mass index; TRV, tricuspid regurgitation velocity; LVEF, left ventricular ejection fractions; RAD, right atrium diameter; PASP, pulmonary artery systolic pressure; RVIDd, right ventricular internal diameter at end-diastole; LVIDd, left ventricular internal diameter at end-diastole; RV/LV, Right ventricle/left ventricle basal diameter ratio. ** *p* < 0.01 versus controls; ^#^ Peak TRV and PASP were measured for 12 controls, 7 smokers, and 12 smokers with PH whose tricuspid regurgitation (TR) could be found by echocardiography.

## Data Availability

Data will be made available on request.

## Supplementary Materials

The following supporting information can be downloaded at: https://www.mdpi.com/article/10.3390/toxics12060396/s1, Figure S1: The correlation between miR-21 levels and PASP, markers of endothelial dysfunction and markers of cell senescence in lung tissues from patients; Figure S2: CS exposure induces right-heart hypertrophy and pulmonary vascular proliferation in mice; Figure S3: CSE promotes the senescence of HUVECs; Figure S4: Downregulation of miR-21 alleviated the senescence of HUVECs induced by CSE; Figure S5: Knockout of miR-21 alleviates right-heart hypertrophy and pulmonary vascular proliferation induced by CS exposure in mice.
