# Supplementary material for: miR-21-Mediated Endothelial Senescence and Dysfunction Are Involved in Cigarette Smoke-Induced Pulmonary Hypertension through Activation of PI3K/AKT/mTOR Signaling
Source: Toxics. 2024 May 29;12(6):396. doi: 10.3390/toxics12060396 (PMC11209295; doi:10.3390/toxics12060396)
Supplement: Supplementary file 1 [file toxics-12-00396-s001.zip › toxics-3002527-supplementary.pdf]

Supplementary Materials

miR-21-Mediated Endothelial Senescence and Dysfunction Are Involved in Cigarette Smoke-Induced Pulmonary Hypertension through Activation of PI3K/AKT/mTOR Signaling

Bin He [1,2,†], Binxia Shao [2,3,†], Cheng Cheng [2,†], Zitong Ye [2], Yi Yang [2], Bowen Fan [2], Haibo Xia [2], Hao Wu [1], Qizhan Liu [2,*] and Jinsong Zhang [1,*]

1 Department of Emergency, Jiangsu Province Hospital, The First Affiliated Hospital of Nanjing Medical University, Nanjing 210029, China; hbhbwywy34@sina.com (B.H.); drwuhao@njmu.edu.cn (H.W.)
2 The Key Laboratory of Modern Toxicology, Ministry of Education, School of Public Health, Nanjing Medical University, Nanjing 211166, China; shaobinxia@126.com (B.S.); chengc1003@163.com (C.C.); 19190112@stu.njmu.edu.cn (Z.Y.); njmuyy@outlook.com (Y.Y.); fanbowen0908@163.com (B.F.); haiboxia346@163.com (H.X.)
3 Department of Emergency, Nanjing Drum Tower Hospital, The Affiliated Hospital of Medical School of Nanjing University, Nanjing 210008, China
* Correspondence: drqzliu@hotmail.com (Q.L.); zhangjso@njmu.edu.cn (J.Z.)
† These authors contributed equally to this work.

Contents of supplementary information

Supplementary figures.

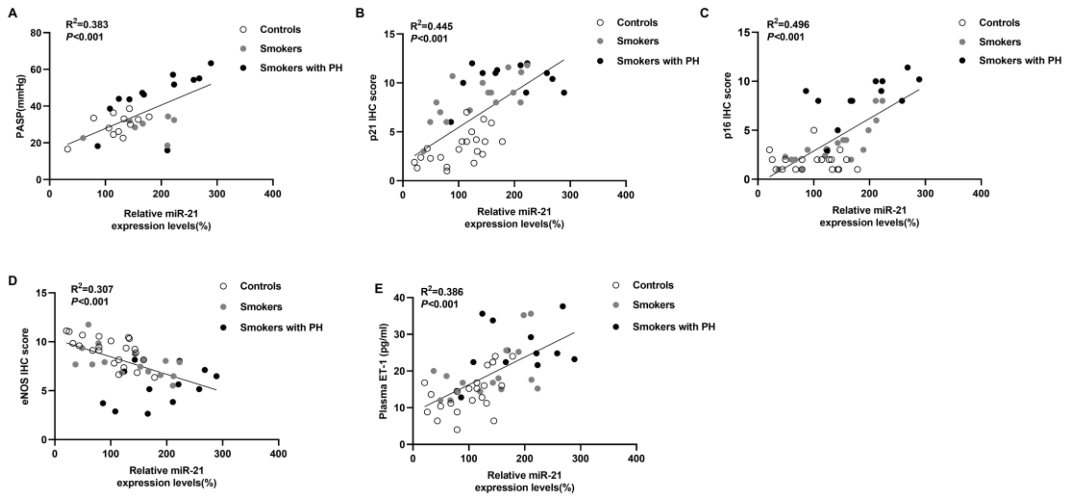

Figure S1. The correlation between miR-21 levels and PASP, markers of endothelial dysfunction and markers of cell senescence in lung tissues from patients

(A) miR-21 levels in lung tissues positively correlate with pulmonary artery systolic pressure (PASP). Only 31 patients (12 controls, 7 smokers, and 12 smokers with PH) were utilized, since their tricuspid regurgitation velocity (TRV) was measurable, and PASP could be calculated. (B, C) miR-21 levels positively correlate with IHC scores of p21- and p16-positive endothelial cells (ECs) in lung tissues from patients. (D) Upregulation of miR-21 levels correlates with decreased eNOS expression in lung tissues, and (E) raised plasma ET-1 levels of patients, related to miR-21 expression.

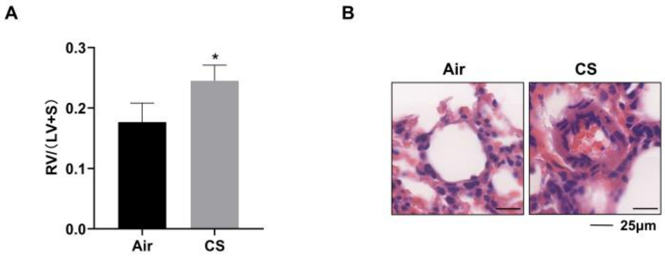

Figure S2. CS exposure induces right-heart hypertrophy and pulmonary vascular proliferation in mice.

(A) The degree of RV hypertrophy, given as the ratio of RV to the left ventricular plus septum (LV+S) mass was calculated for mice. (B) H&E staining of small pulmonary vessels (diameter: 0-70 μm) from mice (bars: 25 μm). * $p < 0.05$ compared with mice exposed to air.

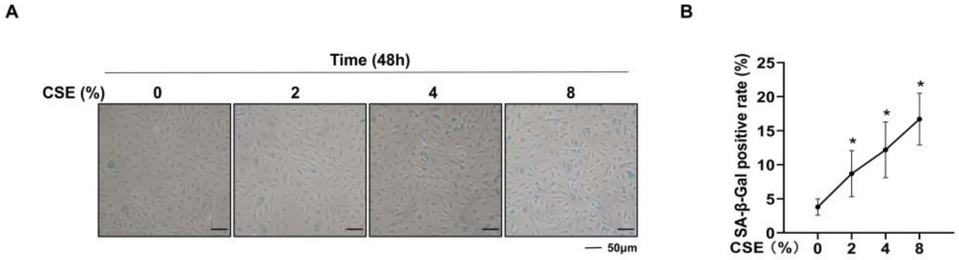

Figure S3. CSE promotes the senescence of HUVECs.

(A) Representative SA-β-gal staining images of cells (blue-stained positive cells) and (B) the percentage of positive cells (n = 3, Scale bars: 50 μm). * $p < 0.05$ different from HUVECs not treated with CSE.

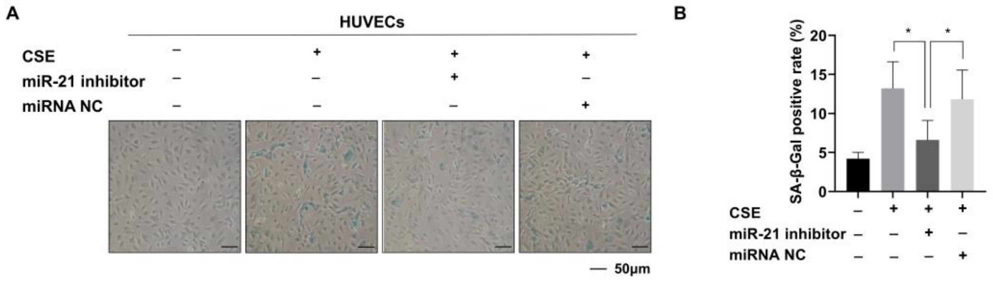

Figure S4. Downregulation of miR-21 alleviated the senescence of HUVECs induced by CSE.

(A) Representative SA-β-gal staining images of cells (blue-stained positive cells) by using the miR-21 inhibitor. (B) the percentages of positive cells (n = 3, Scale bars: 50 μm). * $p < 0.05$ different from HUVECs treated with 8% CSE and miR-21 inhibitor.

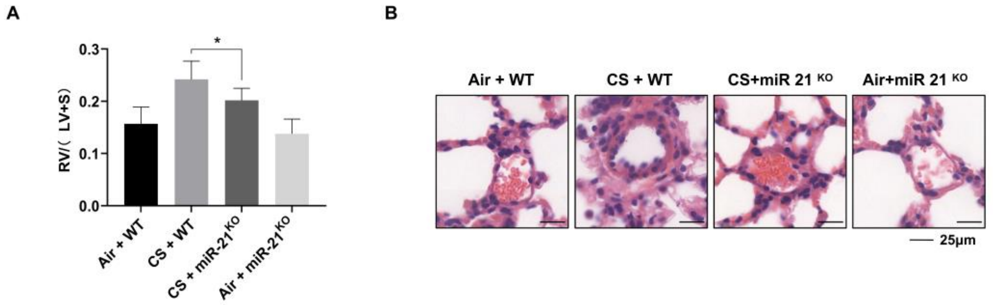

Figure S5. Knockout of miR-21 alleviates right-heart hypertrophy and pulmonary vascular proliferation induced by CS exposure in mice.

(A) The ratios of RV and the left ventricular plus septum (LV+S) mass were calculated for mice. (B) H&E staining of small pulmonary vessels (diameter: 0-70 μm) from mice (bars: 25 μm). * $p < 0.05$ compared with WT mice exposed to CS.
